# External validation suggests Integrin beta 3 as prognostic biomarker in serous ovarian adenocarcinomas

**DOI:** 10.1186/1471-2407-9-336

**Published:** 2009-09-23

**Authors:** Karolina Partheen, Kristina Levan, Lovisa Österberg, Ingela Claesson, Karin Sundfeldt, György Horvath

**Affiliations:** 1 Department of Oncology, Institute of clinical sciences, University of Gothenburg, Gothenburg, Sweden; 2 Department of Obstetrics and Gynecology, Institute of clinical sciences, University of Gothenburg, Gothenburg, Sweden

## Abstract

**Background:**

The majority of women with ovarian cancer are diagnosed in late stages, and the mortality rate is high. The use of biomarkers as prognostic factors may improve the treatment and clinical outcome of these patients. We performed an external validation of the potential biomarkers CLU, ITGB3, CAPG, and PRAME to determine if the expression levels are relevant to use as prognostic factors.

**Methods:**

We analysed the gene expression of CLU, ITGB3, CAPG, and PRAME in 30 advanced staged serous adenocarcinomas with quantitative real-time polymerase chain reaction (QPCR) and the protein levels were analysed in 98 serous adenocarcinomas with western blot for semiquantitative analysis. Statistical differences in mRNA and protein expressions between tumours from survivors and tumours from deceased patients were evaluated using the Mann-Whitney U test.

**Results:**

The gene and protein ITGB3 (Integrin beta 3) were significantly more expressed in tumours from survivors compared to tumours from deceased patients, which is in concordance with our previous results. However, no significant differences were detected for the other three genes or proteins CLU, CAPG, and PRAME.

**Conclusion:**

The loss of ITGB3 expression in tumours from deceased patients and high expression in tumours from survivors could be used as a biomarker for patients with advanced serous tumours.

## Background

Ovarian carcinoma is the fifth most common cause of cancer death among women in Western Europe and the United states [1,2]. The most common type is epithelial ovarian cancer, which has several histopathological subtypes including serous, mucinous, endometrioid, and clear cell carcinomas. Among these, serous papillary adenocarcinoma is the most common form accounting for approximately 50% of cases [3].

The majority of women with ovarian cancer are diagnosed in late stages, and these patients are treated similarly with tumour reductive surgery followed by chemotherapy. However, even though patients with stage III-IV tumours undergo extensive therapy, the mortality rate is high, and only 30% of patients survive more than five years after diagnosis [4]. The prognostic factors used today, such as surgical stage, volume of residual tumour after primary surgery, and histologic grade, are insufficient to optimise and individualise the treatment. There is an urgent need to better classify these tumours to improve the treatment and clinical outcome of patients.

The use of novel biomarkers as prognostic factors may facilitate identification of patients who are likely to relapse and die of the disease. Expression profiles have been used to identify genes involved in ovarian tumour initiation and progression. We have previously used expression arrays to detect potential biomarkers with prognostic relevance [5]. However, there is a limited overlap of data among expression analyses of ovarian carcinomas owing to several factors [6]. First, the number of tumours studied is low and consists of different stages and mixtures of histopathological subtypes. Second, different microarray technology platforms and statistics are used, and the definition of different endpoints varies in the studies. The extensive knowledge about differences in microarray results suggests that external validations are required to verify if the data are relevant to use. Our previous analysis demonstrated differences in expression between tumours from survivors and tumours from deceased patients, including four cancer-related genes, *ITGB3*, *CLU*, *CAPG*, and *PRAME *[5]. We further established the significant differences in expression levels for the corresponding proteins [7]. Further validation of the gene and protein expressions in an external validation set is necessary, to clarify if the expression levels are usable as biomarkers. The protein expressions of these potential biomarkers may also be related to the tumours biological properties.

Integrin beta 3 (ITGB3) was more expressed in tumours from survivors in our previous studies. Integrins are known to participate in cell adhesion, and work as receptors in cell-surface mediated signalling, through binding with different ligands in focal adhesions [8-10]. Clusterin (CLU), also more expressed in tumours from survivors in our previous studies, seems to have divergent functions in cells. This is probably due to different isoforms, one secreted/cytoplasmic form (sCLU/cCLU) with chaperone activity involved in tumour progression, and one nuclear form (nCLU) with proapoptotic function [11-13]. Capping protein (actin filament) gelsolin-like (CAPG), which was more expressed in tumours from deceased patients in our studies, belongs to the gelsolin protein superfamily. This group of proteins control actin organisation, and CAPG contributes to the control of actin-based motility in cells by capping the barbed ends of actin filaments [14,15]. Preferentially expressed antigen in melanoma (PRAME) is a repressor of retinoic acid receptor signalling [16]. In our previous studies, PRAME was detected as more expressed in tumours from deceased patients compared to tumours from survivors.

In this study, we performed an external validation of the four genes and proteins in a new set of advanced ovarian serous adenocarcinomas to determine if differences in expression levels are relevant to use as prognostic markers.

## Methods

### Patient and tumour material

In this study, 98 advanced stage (III or IV) serous papillary adenocarcinomas of the ovary were analyzed (Table 1). The tumours were randomly selected and adjusted to similar size of tumours from survivors and tumours from deceased patients. Patients who survived five years or more after the initial diagnosis were considered as survivors and all deceased patients in the study succumbed to cancer. Surgical staging of the tumours was performed according to International Federation of Gynaecology and Obstetrics (FIGO) standards, and patients with no macroscopic residual tumour were classified as radically operated. The tumours were removed at primary surgery and secured for pathology examination, RNA, and protein extraction. After surgery, patients were treated with platinum based chemotherapy, either with a combination of farmorubicine, carboplatin, and cyclophosphamide, or with a combination of paclitaxel and carboplatin. The tumours were collected from patients diagnosed between 1993 and 2003 at Sahlgrenska University Hospital, Gothenburg, Sweden, and the study was approved by the local ethics committee. A pathologist reviewed all tumours according to the treatment protocol for gynaecological malignancies in western Sweden. Specimen imprints for cytologic evaluation were performed to verify the presence of tumour cell content, and only tumours containing at least 50% tumour cells were included.

**Table 1:** Summary of clinicopathologic characteristics of the patients

	Tumours (from survivors/deceased patients)
**Total Tumours**	98 (48/50)

**Tumours used in QPCR analysis**	30 (15/15)

**Mean age **(years)	60 (57/62) (range 28-81)

**FIGO Staging**	
IIIa	10 (7/3)
IIIb	14 (7/7)
IIIc	73 (33/40)
IV	1 (1/0)

**Surgery**	
Radically operated	20 (13/7)
Residual tumour	75 (34/41)
Not available	3 (1/2)

**Differentiation**	
Well	15 (10/5)
Moderate	22 (10/12)
Poor	59 (26/33)
Not available	2 (2/0)

**Treatment**	
farmorubicine, carboplatin and cyclophosphamide	31 (16/15)
paclitaxel and carboplatin	67 (32/35)

### Quantitative real-time polymerase chain reaction (QPCR)

Thirty tumours from patients treated with paclitaxel and carboplatin were analysed with QPCR, performed as described by Partheen et al. [7]. Briefly, total RNA was isolated from frozen tumours by homogenisation with TRIzol Reagent (Invitrogen, Carlsbad, CA, USA) and then extracted with RNeasy mini kit (Qiagen, Valencia, CA, USA). High-quality RNA was obtainable from 30 samples and used in the analysis. From each tumour sample 0.5 μg of total RNA was reverse transcribed in duplicate. Each cDNA sample was analysed once by real-time PCR, giving two data points for each tumour sample. The cDNA were detected with SYBR Green I. Two stable reference genes were used for normalisation, GAPDH and β-actin from the Human Endogenous Control Gene Panel (TATAA Biocenter, http://www.tataa.com). The efficiency of each QPCR assay was estimated from the slope of a standard curve generated from the serial dilution of purified PCR products. The assays for CLU and ITGB3 showed PCR efficiencies close to 80%, and for CAPG and PRAME 90%. These values were used for subsequent calculations. For each assay the average Ct value for each tumour sample was converted to relative copy numbers. The data were then normalised by the geometric average of the two reference genes [17].

### Western blot

Western blot was performed on 98 tumour samples, each in duplicate as described by Partheen et al. [7]. Briefly, the frozen samples were homogenised with RIPA followed by centrifugation. The samples were diluted in SDS sample buffer with and without 10% 2-mercaptoethanol and heated at 97°C for 5 minutes. The unreduced samples (without 2-mercaptoethanol) were used to detect ITGB3. Total protein was loaded into each lane on a 10% Criterion™ precast gel (BIO-RAD laboratories, Hercules, CA, USA). The proteins were transferred to nitrocellulose membranes (BIO-RAD) and blocked overnight at 4°C in 5% non-fat milk in 10 mM Tris buffered saline (TBS).

The membranes were incubated in TBS containing 0,05%Tween 20 with the following primary antibodies: chicken polyclonal to CAPG (1:3000, ab14235, Abcam, Cambridge, UK), rabbit polyclonal to PRAME (1:1000, ab32185, Abcam), mouse monoclonal to CLU (1:15 000, clone 41D, Upstate Biotechnology, Lake Placid, NY, USA), ITGB3 (1:1000, MAB1974, Chemicon, Temecula, CA, USA) and GAPDH as a relative loading control (1:15 000, ab8245, Abcam). Proteins were visualised by chemiluminescence, using horseradish peroxidase-linked (HRP) secondary antibodies; goat anti-rabbit (1:5000, ab6721, Abcam), anti-chicken (1:3000, GAYFC-HRP, Genway Biotech, San Diego, CA, USA) and anti-mouse (1:5000, sc-2005, Santa Cruz Biotechnology Inc). The membranes were exposed to Amersham Hyperfilm™ ECL (Amersham, Buckinghamshire, UK). The optical density from each band was measured using the software package Quantity One (BIO-RAD) and used for semiquantitative analysis of the proteins. The CLU antibody used detects all isoforms of the protein, and the mature form of CLU at approximately 40 kDa was analysed in this study [18]. An internal reference sample containing pooled protein from ovarian tumours, same on each blot, was used as a standard for quantification and was given the value 1. The mean value of the duplicates was calculated and used in the analysis.

### Statistical analysis

Statistical analysis of the data was performed using SPSS for Windows (version 12.0.1). The tumours used in the protein analysis were from patients treated with slightly different carboplatin-based chemotherapies. To check if the two treatment groups could be combined in the statistical analysis, treatment was tested as a 2-level factor, as well as an interaction with protein expression in logistic regression models with survival as response. Due to the skewed expression distributions, the logarithms of protein expressions were used as explanatory variables in the model, and nonparametric tests were used in the analysis of statistical differences.

Statistical differences in mRNA and protein expressions between tumours from survivors and tumours from deceased patients were evaluated using the Mann-Whitney U test. Logistic regression was performed on logarithmic values of protein expressions and the prognostic factors age, stage and surgery outcome to define if these factors contributes significantly to the prediction of survival. The relation between expressions measured with QPCR and western blot was evaluated with bivariate correlation using Spearman correlation coefficient. We used Kaplan-Meier survival curves to show differences in clinical outcome between patients with tumours that expressed high alternatively low levels of ITGB3 mRNA or protein, using cut-off values at 0.29 and 0.43 respectively, based on the detected relative expression levels. A log-rank test was used to compare the curves. A value of *p *< 0.05 was considered to be significant.

## Results

### The gene ITGB3 was detected as differently expressed

**Figure 1:**
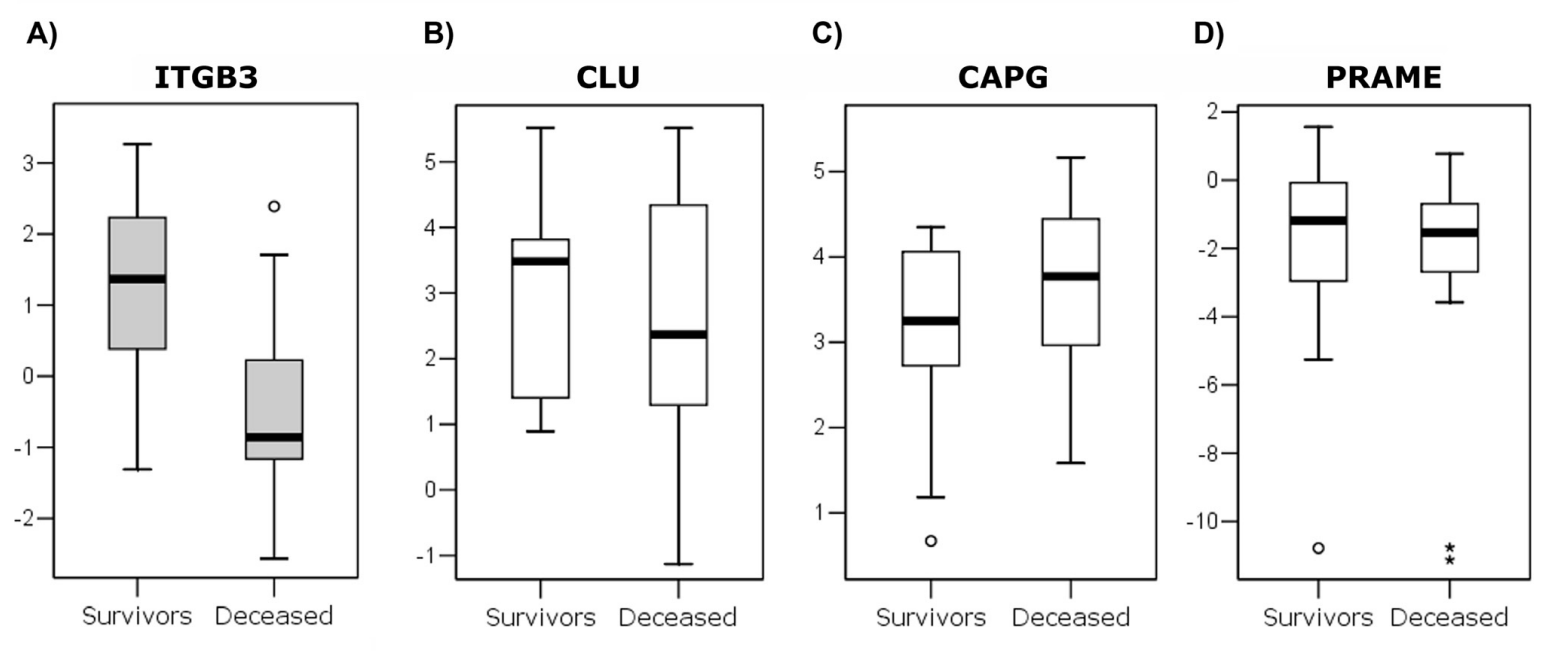
**Relative mRNA copy numbers for the genes**. Relative mRNA copy numbers as log2 values for the genes A) ITGB3 (significantly differently expressed, *p *= 0.004) B) CLU C) CAPG and D) PRAME. Each plot shows the medians (centre lines), interquartile ranges (boxes), largest and smallest values (whiskers) that are not outliers (circles), or extreme values (stars) within a category.

The expression of *CLU*, *ITGB3*, *CAPG*, and *PRAME *was investigated to study differences in expression levels between tumours from survivors and tumours from deceased patients. The four genes were analysed with QPCR in 30 of the 98 serous adenocarcinomas from patients treated with paclitaxel and carboplatin. The number of tumours analysed was reduced, because the RNA quality did not reach the desirable level in 68 samples. The expression in tumours from survivors and in tumours from deceased patients were compared, and *ITGB3 *was significantly more expressed in tumours from survivors (*p *= 0.004) (Figure 1). The best separation between the two groups was obtained with a cut-off value of the relative expression at 0.29 (Figure 1A). Only three tumours from survivors expressed *ITGB3 *less than 0.29 and three tumours from deceased patients more than 0.29. No statistical differences were detected for the other three genes analysed: *CAPG *(*p *= 0.24), *CLU *(*p *= 0.76), and *PRAME *(*p *= 0.52).

### The protein ITGB3 was detected as differently expressed

**Figure 2:**
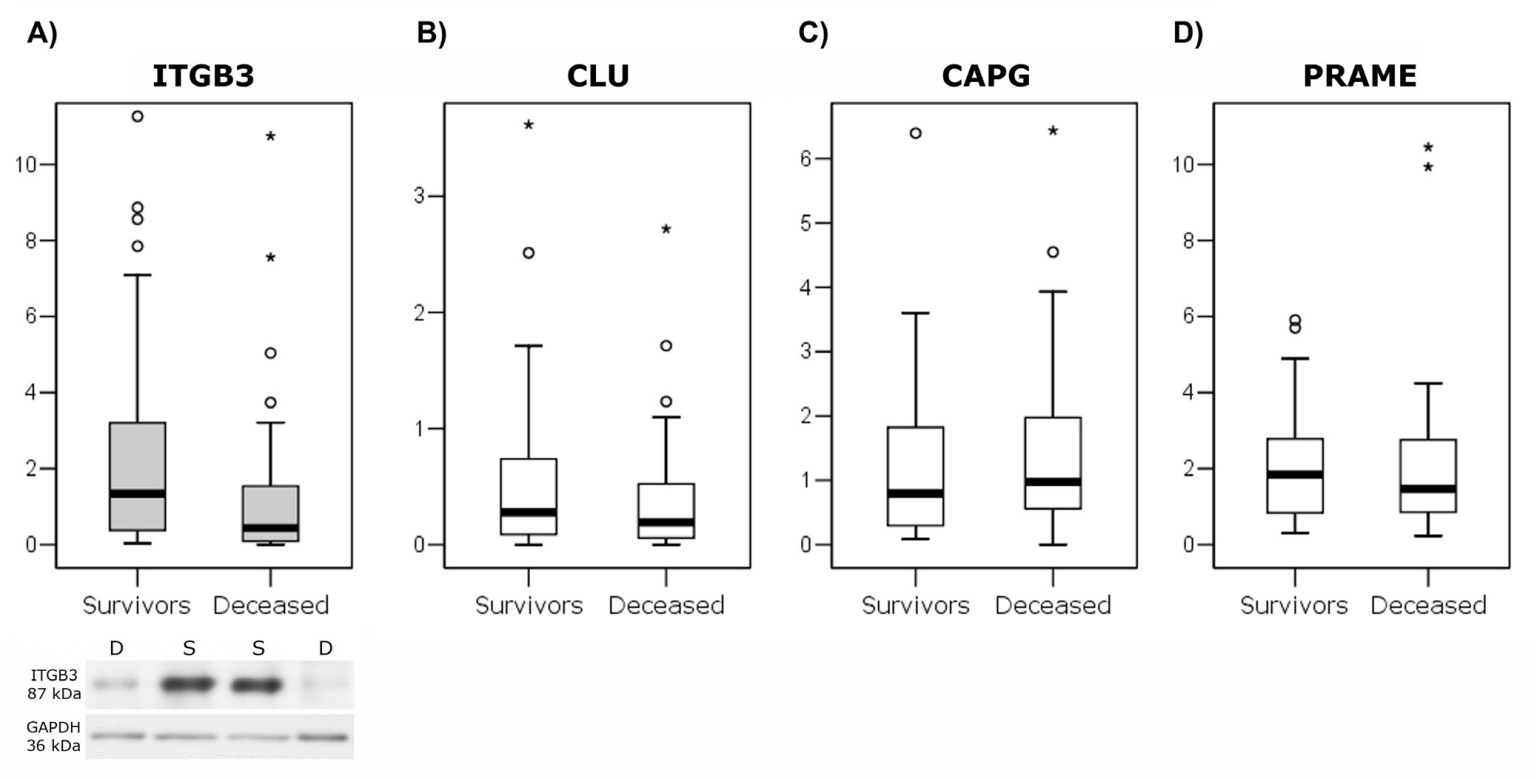
**Relative protein expression for the proteins**. Relative protein expression of A) ITGB3^a ^(significantly differently expressed, *p *= 0.005) B) CLU C) CAPG and D) PRAME. Each plot shows the medians (centre lines), interquartile ranges (boxes), largest and smallest values (whiskers) that are not outliers (circles), or extreme values (stars) within a category. A representative immunoblot is shown for ITGB3, where GAPDH was used as loading control and tumours from survivors (S) and deceased (D) patients are indicated. ^a^In the figure, ITGB3 values for two survivors and one deceased patients were excluded due to extremely high values.

The proteins CLU, ITGB3, CAPG, and PRAME were examined with western blot for semiquantitative measurements of each protein in 98 tumours (Figure 2). Type of patient treatment could be ignored in the analysis, since no significant effects on protein expressions were detected. A significant difference in expression between tumours from survivors and tumours from deceased patients was detected for ITGB3 (*p *= 0.005), but not for the other proteins, CLU (*p *= 0.15), PRAME (*p *= 0.62), and CAPG (*p *= 0.24). The most optimal cut-off value for ITGB3 that separated survivors and deceased patients turned out to be 0.43 (logarithmic value -0.87). The logistic regression analysis using age, stage, surgery outcome and protein expressions showed that ITGB3 (*p *= 0.002) and survival (*p *= 0.013) was independent factors to predict survival. In addition, significant differences of ITGB3 expression were also detected when tumours were divided according to treatment, with *p *= 0.05 for paclitaxel and carboplatin treated patients and *p *= 0.04 for farmorubicine-, carboplatin-, and cyclophosphamide-treated patients.

**Figure 3:**
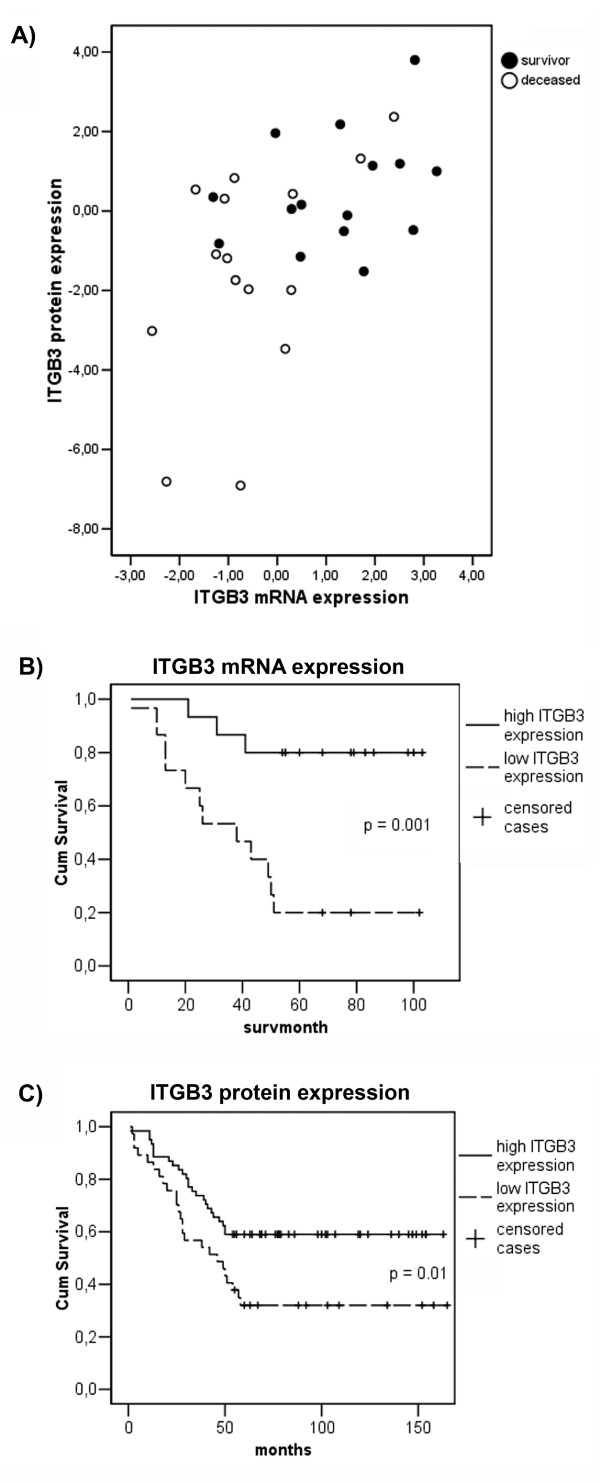
**Visualization of differences in ITGB3 expression levels between survivors and deceased patients**. A) The logarithmic relative values of protein and mRNA expressions from the same tumour plotted against each other. B) Kaplan-Meier survival curve for ITGB3 mRNA and C) the protein ITGB3.

The relative expression of ITGB3 mRNA and protein correlated well (*p *= 0.027). Both mRNA and protein expressions at low levels are dominated by tumours from deceased patients, as illustrated in Figure 3A. Kaplan-Meier survival curves for high versus low expression of ITGB3 mRNA and protein are shown in Figure 3B and 3C, respectively. There was a significant difference in survival for tumours with high versus low expression of the mRNA and protein.

## Discussion

In this study, we performed an external validation of four potential prognostic biomarkers, ITGB3, CLU, CAPG, and PRAME, in advanced ovarian serous adenocarcinomas. We compared the gene and protein expression levels between tumours from survivors and tumours from deceased patients, and ITGB3 was the one found as differently expressed. The clinical outcome of patients with advanced ovarian adenocarcinoma is difficult to predict at an individual level, and ITGB3 could be a complementary potential biomarker for patients with serous tumours. This is of importance because an improvement of the prognostic factors may make it possible to optimise patients' treatment. The differences were detected for both the gene and the protein expressions, which strengthens the credibility of our results.

A limitation in many studies of biomarkers, with microarray and other methods, is the lack of follow-up with external validation of significant findings. Consequently, the insignificant results for CLU, CAPG, and PRAME in the present study highlight the importance of verifying data in an external validation set of tumours, although, the number of tumours analysed with QPCR in this study was low, and small groups may have had an impact on the results. However, earlier studies have reported CLU as down-regulated and CAPG and PRAME as up-regulated in advanced carcinomas compared to early tumours or normal tissue [19-25]. Even though we did not detect any differences in relation to survival in our analysis, the expression of CLU, CAPG, and PRAME might still be associated with the development and progression of serous ovarian adenocarcinomas. Concerning ITGB3, an evaluation in the reverse order, where the patients are classified depending on the tumours' ITGB3 expression, could determine if it is possible to distinguish patients who will respond to standard treatment from those who will not.

The gene *ITGB3 *encodes the beta chain of two integral cell-surface glycoproteins, αvβ3 and GPIIb/IIIa, of which αvβ3 is expressed on endothelial and tumour cells [26]. As molecules involved in cell adhesion and cell signalling, integrins have an important role in various carcinomas. The expression of αvβ3 integrins has been linked to bad prognosis for breast cancer and melanoma patients, but the relation to ovarian tumours needs further clarification [27,28]. The expression of αvβ3 integrins has been detected as less frequent in ovarian epithelial tumours of low malignant potential (LMP) in contrast to ovarian carcinomas [29]. Controversially, Maubant et al. [30] found a significantly less frequent expression of the β3 subunit in grade 3 ovarian tumours compared to grade 1 and 2 tumours, and less expression in peritoneal metastasis compared to primary tumours. Nevertheless, they did not find any correlation to survival, as we did in our study. The use of tumours with different histological subtypes and stages in their analysis may explain the inconsistent findings compared with our results, since we used only advanced serous adenocarcinomas. Further, ITGB3 protein expression have been detected in normal ovarian epithelium and highly differentiated ovarian carcinomas, but are lacking in most of the less-differentiated tumours [31]. Regeneration of normal ovarian epithelial cells is a continuous process due to wound healing after ovulation. This process involves adhesion, spreading, and proliferation of cells, all features that could involve integrins. The expression of ITGB3 in the normal ovarian epithelium and its expression in well-differentiated ovarian carcinomas may reflect the preservation of normal cell properties. The expression of ITGB3 in tumours from survivors in our study may indicate that these tumour cells still retain normal cell properties, and are therefore less aggressive. Moreover, αvβ3 may have different function in various tissues, which may partly explain the divergent results of αvβ3 expression linked to bad prognosis in other cancers, such as breast cancer and melanoma [27,28]. Therefore, the use of ITGB3 as a biomarker may only have clinical relevance in ovarian adenocarcinomas.

Normal adherent cells must be anchored to the extracellular matrix (ECM) to survive and proliferate. Ligated αvβ3 activates cell survival pathways and suppresses proapoptotic signalling, while inactive unligated αvβ3 promotes apoptosis [32,33]. Maubant et al. [34] used a sensitive ovarian cancer cell line and its cisplatin-resistant counterpart to study the expression of αvβ3 and αvβ5. They showed that three days after plating, the αv subunit associated with the β3 subunit on the surface of sensitive cells, but it associated with both β3 and β5 on the surface of resistant cells. Consequently, ITGB3 could have a dual role in cancer. At its primary site, the protein favours a good prognosis, but as soon as the tumour cell has lost its attachment, the cell will be able to metastasise and progress at a distant site. This dual role of the protein may have an impact on the use of ITGB3 as a biomarker.

Finally, the function of ITGB3 as a biomarker in ovarian adenocarcinoma may be influenced by other factors in the tumours. One such factor is the up-regulation of αvβ3 integrin in low HRG/NRG1-expressing breast cancer cell lines [35]. These cell lines are highly sensitive to treatment with paclitaxel. The up-regulation of αvβ3 integrin and low expression of HRG/NRG1 in cancer cells could be a novel molecular marker of chemosensitivity. The correlation of HRG/NRG1 and ITGB3 expression might be relevant to study in ovarian tumours as well, or there might be other unknown factors that regulate the impact of ITGB3 as a biomarker.

## Conclusion

In conclusion, ITGB3 was significantly differently expressed between tumours from survivors and tumours from deceased patients. The differences were detected for both gene and protein levels in our external validation set of advanced serous ovarian adenocarcinomas. The loss of ITGB3 expression in tumours from deceased patients and high expression in tumours from survivors could be used as a biomarker for patients with advanced serous tumours. Prospective evaluation of ITGB3 expression levels could determine if it is possible to distinguish patients who will respond to standard treatment from those who will not. It would also be worth considering a study of ITGB3 expression in stage I and stage II serous ovarian tumours, and in other histopathological subtypes. In future, it may be that patients who respond to standard therapy could be treated with a more moderate combination of anti-cancer agents, and higher-risk patients might be offered additional chemotherapy and more frequent follow up at an initial state.

## Competing interests

The authors declare that they have no competing interests.

## Authors' contributions

KP participated in the conception and design of the study, carried out the western blot, statistical analysis, interpretation of the data and drafted the manuscript. KL and LÖ contributed to the analysis and helped to draft the manuscript. IC carried out the western blot and contributed to the analysis of the data. KS participated in the design, and assisted in the writing of the manuscript. GH contributed to the conception and the design of the study, assisted in the writing of the manuscript, and funded the study. All authors read and approved the final manuscript.

## Pre-publication history

The pre-publication history for this paper can be accessed here:

http://www.biomedcentral.com/1471-2407/9/336/prepub

## References

[B1] Runnebaum IB Stickeler E Epidemiological and molecular aspects of ovarian cancer risk J Cancer Res Clin Oncol 2001 127 2 73 79 10.1007/s004320000153 11216917 PMC12164990

[B2] Socialstyrelsen TSNBoHaW Statistics - Health and Diseases 2007:15. Causes of death, Sweden 2005 2007 The Swedish National Board of Health and Welfare; Sweden

[B3] Fox H Kavanagh JJ, Singletary SE, Einhorn N, DePetrillo AD Pathology of ovarian Cancer Cancer in Women 1998 Oxford: Blackwell Science, Inc 415 442

[B4] Heintz AP Odicino F Maisonneuve P Quinn MA Benedet JL Creasman WT Ngan HY Pecorelli S Beller U Carcinoma of the ovary. FIGO 6th Annual Report on the Results of Treatment in Gynecological Cancer Int J Gynaecol Obstet 2006 95 Suppl 1 S161 192 10.1016/S0020-7292(06)60033-7 17161157

[B5] Partheen K Levan K Osterberg L Horvath G Expression analysis of stage III serous ovarian adenocarcinoma distinguishes a sub-group of survivors Eur J Cancer 2006 42 16 2846 2854 10.1016/j.ejca.2006.06.026 16996261

[B6] Gyorffy B Dietel M Fekete T Lage H A snapshot of microarray-generated gene expression signatures associated with ovarian carcinoma Int J Gynecol Cancer 2008 18 6 1215 1233 10.1111/j.1525-1438.2007.01169.x 18217975

[B7] Partheen K Levan K Osterberg L Claesson I Fallenius G Sundfeldt K Horvath G Four potential biomarkers as prognostic factors in stage III serous ovarian adenocarcinomas Int J Cancer 2008 123 9 2130 2137 10.1002/ijc.23758 18709641

[B8] Nachman RL Leung LL Complex formation of platelet membrane glycoproteins IIb and IIIa with fibrinogen J Clin Invest 1982 69 2 263 269 10.1172/JCI110448 6460044 PMC370974

[B9] Flier A van der Sonnenberg A Function and interactions of integrins Cell Tissue Res 2001 305 3 285 298 10.1007/s004410100417 11572082

[B10] Tamkun JW DeSimone DW Fonda D Patel RS Buck C Horwitz AF Hynes RO Structure of integrin, a glycoprotein involved in the transmembrane linkage between fibronectin and actin Cell 1986 46 2 271 282 10.1016/0092-8674(86)90744-0 3487386

[B11] Michel D Chatelain G North S Brun G Stress-induced transcription of the clusterin/apoJ gene Biochem J 1997 328 Pt 1 45 50 9359832 10.1042/bj3280045 PMC1218885

[B12] Humphreys DT Carver JA Easterbrook-Smith SB Wilson MR Clusterin has chaperone-like activity similar to that of small heat shock proteins J Biol Chem 1999 274 11 6875 6881 10.1074/jbc.274.11.6875 10066740

[B13] Yang CR Leskov K Hosley-Eberlein K Criswell T Pink JJ Kinsella TJ Boothman DA Nuclear clusterin/XIP8, an x-ray-induced Ku70-binding protein that signals cell death Proc Natl Acad Sci USA 2000 97 11 5907 5912 10.1073/pnas.97.11.5907 10823943 PMC18532

[B14] Yin HL Gelsolin: calcium- and polyphosphoinositide-regulated actin-modulating protein Bioessays 1987 7 4 176 179 10.1002/bies.950070409 2825660

[B15] Silacci P Mazzolai L Gauci C Stergiopulos N Yin HL Hayoz D Gelsolin superfamily proteins: key regulators of cellular functions Cell Mol Life Sci 2004 61 19-20 2614 2623 10.1007/s00018-004-4225-6 15526166 PMC11924436

[B16] Epping MT Wang L Edel MJ Carlee L Hernandez M Bernards R The human tumor antigen PRAME is a dominant repressor of retinoic acid receptor signaling Cell 2005 122 6 835 847 10.1016/j.cell.2005.07.003 16179254

[B17] Vandesompele J De Preter K Pattyn F Poppe B Van Roy N De Paepe A Speleman F Accurate normalization of real-time quantitative RT-PCR data by geometric averaging of multiple internal control genes Genome Biol 2002 3 7 RESEARCH0034 10.1186/gb-2002-3-7-research0034 12184808 PMC126239

[B18] Pucci S Bonanno E Pichiorri F Angeloni C Spagnoli LG Modulation of different clusterin isoforms in human colon tumorigenesis Oncogene 2004 23 13 2298 2304 10.1038/sj.onc.1207404 14755245

[B19] Jhala N Jhala D Vickers SM Eltoum I Batra SK Manne U Eloubeidi M Jones JJ Grizzle WE Biomarkers in Diagnosis of pancreatic carcinoma in fine-needle aspirates Am J Clin Pathol 2006 126 4 572 579 10.1309/CEV30BE088CBDQD9 17019794

[B20] Gilks CB Vanderhyden BC Zhu S Rijn M van de Longacre TA Distinction between serous tumors of low malignant potential and serous carcinomas based on global mRNA expression profiling Gynecol Oncol 2005 96 3 684 694 10.1016/j.ygyno.2004.11.039 15721412

[B21] Bettuzzi S Davalli P Astancolle S Carani C Madeo B Tampieri A Corti A Tumor progression is accompanied by significant changes in the levels of expression of polyamine metabolism regulatory genes and clusterin (sulfated glycoprotein 2) in human prostate cancer specimens Cancer Res 2000 60 1 28 34 10646846

[B22] Van Ginkel PR Gee RL Walker TM Hu DN Heizmann CW Polans AS The identification and differential expression of calcium-binding proteins associated with ocular melanoma Biochim Biophys Acta 1998 1448 2 290 297 10.1016/S0167-4889(98)00133-5 9920419

[B23] Ikeda H Lethe B Lehmann F van Baren N Baurain JF de Smet C Chambost H Vitale M Moretta A Boon T Coulie PG Characterization of an antigen that is recognized on a melanoma showing partial HLA loss by CTL expressing an NK inhibitory receptor Immunity 1997 6 2 199 208 10.1016/S1074-7613(00)80426-4 9047241

[B24] Oberthuer A Hero B Spitz R Berthold F Fischer M The tumor-associated antigen PRAME is universally expressed in high-stage neuroblastoma and associated with poor outcome Clin Cancer Res 2004 10 13 4307 4313 10.1158/1078-0432.CCR-03-0813 15240516

[B25] Thompson CC Ashcroft FJ Patel S Saraga G Vimalachandran D Prime W Campbell F Dodson A Jenkins RE Lemoine NR Crnogorac-Jurcevic T Yin HL Costello E Pancreatic cancer cells overexpress gelsolin family-capping proteins, which contribute to their cell motility Gut 2007 56 1 95 106 10.1136/gut.2005.083691 16847067 PMC1856675

[B26] Thiagarajan P Shapiro SS Levine E DeMarco L Yalcin A A monoclonal antibody to human platelet glycoprotein IIIa detects a related protein in cultured human endothelial cells J Clin Invest 1985 75 3 896 901 10.1172/JCI111789 3156882 PMC423621

[B27] Rolli M Fransvea E Pilch J Saven A Felding-Habermann B Activated integrin alphavbeta3 cooperates with metalloproteinase MMP-9 in regulating migration of metastatic breast cancer cells Proc Natl Acad Sci USA 2003 100 16 9482 9487 10.1073/pnas.1633689100 12874388 PMC170944

[B28] Felding-Habermann B Fransvea E O'Toole TE Manzuk L Faha B Hensler M Involvement of tumor cell integrin alpha v beta 3 in hematogenous metastasis of human melanoma cells Clin Exp Metastasis 2002 19 5 427 436 10.1023/A:1016377114119 12198771

[B29] Liapis H Adler LM Wick MR Rader JS Expression of alpha(v)beta3 integrin is less frequent in ovarian epithelial tumors of low malignant potential in contrast to ovarian carcinomas Hum Pathol 1997 28 4 443 449 10.1016/S0046-8177(97)90033-2 9104944

[B30] Maubant S Cruet-Hennequart S Dutoit S Denoux Y Crouet H Henry-Amar M Gauduchon P Expression of alpha V-associated integrin beta subunits in epithelial ovarian cancer and its relation to prognosis in patients treated with platinum-based regimens J Mol Histol 2005 36 1-2 119 129 10.1007/s10735-004-4273-0 15704006

[B31] Carreiras F Denoux Y Staedel C Lehmann M Sichel F Gauduchon P Expression and localization of alpha v integrins and their ligand vitronectin in normal ovarian epithelium and in ovarian carcinoma Gynecol Oncol 1996 62 2 260 267 10.1006/gyno.1996.0225 8751559

[B32] Frisch SM Francis H Disruption of epithelial cell-matrix interactions induces apoptosis J Cell Biol 1994 124 4 619 626 10.1083/jcb.124.4.619 8106557 PMC2119917

[B33] Meredith JE Jr Fazeli B Schwartz MA The extracellular matrix as a cell survival factor Mol Biol Cell 1993 4 9 953 961 8257797 10.1091/mbc.4.9.953 PMC275725

[B34] Maubant S Cruet-Hennequart S Poulain L Carreiras F Sichel F Luis J Staedel C Gauduchon P Altered adhesion properties and alphav integrin expression in a cisplatin-resistant human ovarian carcinoma cell line Int J Cancer 2002 97 2 186 194 10.1002/ijc.1600 11774263

[B35] Vellon L Menendez JA Liu H Lupu R Up-regulation of alphavbeta3 integrin expression is a novel molecular response to chemotherapy-induced cell damage in a heregulin-dependent manner Differentiation 2007 75 9 819 830 10.1111/j.1432-0436.2007.00241.x 17999741

